# Mercury and Organic Pollutants Removal from Aqueous Solutions by Heterogeneous Photocatalysis with ZnO-Based Materials

**DOI:** 10.3390/molecules28062650

**Published:** 2023-03-15

**Authors:** Elisa Gaggero, María José López-Muñoz, Maria Cristina Paganini, Amaya Arencibia, Stefano Bertinetti, Nieves Fernández de Paz, Paola Calza

**Affiliations:** 1 Department of Chemistry, Università degli Studi di Torino, 10125 Torino, Italy; 2 Departamento de Tecnología Química y Ambiental, ESCET, Universidad Rey Juan Carlos, 28933 Móstoles, Madrid, Spain; 3 Departamento de Tecnología Química, Energética y Mecánica, ESCET, Universidad Rey Juan Carlos, 28933 Móstoles, Madrid, Spain; 4 Metrohm Hispania, 28044 Madrid, Spain

**Keywords:** contaminants of emerging concern, photocatalysis, zinc oxide, mercury removal, aquaculture, geosmin, 2-methylisoborneol

## Abstract

The removal of four Contaminants of Emerging Concern, namely bisphenol A, sulfamethoxazole, diclofenac and benzotriazole; two odorous compounds, geosmin and 2-methylisoborneol, frequently detected in recirculating aquaculture systems; and Hg(II) was investigated using ZnO-based materials doped or co-doped with Ce and Cu under simulated solar radiation. Photocatalysts were synthetized via a hydrothermal route and their efficiency was assessed by changing some operational parameters in different water matrices of increasing complexity. The mixture of contaminants was successfully degraded in just 1 h, while the complete mineralization was achieved in a few hours; experiments performed in an actual aquaculture water confirmed the efficiency and broad versatility of the synthesized materials.

## 1. Introduction

Human health, conservation of the ecosystems and biodiversity and socioeconomic development strongly depend on water quality. In the last century, freshwater consumption has increased remarkably. According to the World Health Organization (WHO), more than 13,000 people die every day due to the onset of diseases related to water unavailability or contamination [1,2]. Climate change also contributes to the water crisis by altering water flows, with the increase in rainfall and extreme meteorological phenomena in some geographical areas in contrast to long periods of drought in other regions. Moreover, urbanization, economic development and the increase in industrial production, especially in the pharmaceutical, electronic and energy sectors, results in a considerable amount of urban and industrial wastewater which, combined with agricultural activities, degrades the quality of water and leads to the pollution of many water resources worldwide [3]. In particular, potentially toxic elements (PTEs) and contaminants of emerging concern (CECs) are conveyed to the environment by multiple routes, making targeted actions to treat contaminated water essential [4,5]. The term CECs includes a wide range of organic molecules, such as pharmaceuticals and personal care products, artificial sweeteners, endocrine-disrupting compounds, etc. The list of compounds belonging to this registry of recalcitrant pollutants is constantly evolving and, consequently, most of them are not yet regulated by legislation due to the lack of exhaustive data on toxicity, mode of action, bioaccumulation capacity and reactivity. Most of these compounds share an incomplete removal during the conventional wastewater treatment plant process as the treatment plants were not designed to treat these kind of pollutants, and as a result they are discharged into the environment [6,7]. Conversely, the toxicology and the related environmental problems of PTEs such as arsenic, mercury, lead and cadmium are in most cases well known so that many regulations governing their release into the environment exist; PTEs are ubiquitous and persistent and may be toxic to biota even at low concentrations. Despite this, many industrial activities such as mining operations, electroplating industries, metal plating activities, and especially battery manufacturing, release PTEs into the waste produced and, if not properly treated, into the environment. They are inherently non-biodegradable and can bioaccumulate in fish and biomagnify along the food chain with resultant negative health effects in humans [8,9]. As a consequence, the increasing presence of PTEs in water systems has not only affected the quality of the water but has also influenced the food sector including the aquaculture industry.

In the last few decades, aquaculture has been the fastest growing food-producing sector worldwide and it is expected to be the only way to meet the increasing demand for aquatic food in the future. In this context, recirculating aquaculture systems became a valid alternative to traditional open systems thanks to the higher production per unit of surface area and the lower needs of land and water [10,11]. However, one of the main drawbacks in these systems is the accumulation of unpleasant tastes and odors in the fish meat: the most common are earthy and moldy ones, mainly caused by two saturated bicyclic terpenoids, geosmin and 2-methylisoborneol (MIB), semi-volatile compounds that can be absorbed by fish and accumulate in lipid-rich tissues. Although they are not toxic to humans at the concentrations usually detected, they can be perceived by our senses even at extremely low concentrations and their presence can make fish unsuitable for commercialization and human consumption, so their removal becomes essential [12,13]. In addition, water used for aquaculture can also be subject to incoming contamination by CECs and PTEs with the subsequent negative impact on the quality of the fish produced. Therefore, finding and developing efficient methods with a low environmental impact for the removal of a wide range of pollutants is of great usefulness and importance. Within this framework, in the last few decades, advanced oxidation processes and heterogeneous photocatalysis based on transition metal oxide semiconductor nanomaterials have been widely developed, finding application in the abatement of contaminants of emerging concern [14,15,16,17,18,19] and in the removal and recovery of a variety of metal ions from aqueous matrices [20,21]. However, the photocatalytic efficiency of the most used semiconductors is limited by their very poor response to visible light; hence, constraining their use in water treatment plants and the sustainability of the process [22]. Different strategies such as anion and cation doping [23,24,25], surface deposition [26,27] and instauration of heterojunctions [28,29] have been developed aimed to efficiently exploit the use of sunlight.

In the present work, the abatement of a mixture of a broad spectrum of pollutants by means of hydrothermally synthetized pristine ZnO, CeO_2_/ZnO and Cu_2_O/CeO_2_/ZnO photocatalysts was investigated. As studied and described in previous works [30,31,32], the interface CeO_2_-ZnO allows the stabilization in the solid of a larger amount of photoinduced charge carriers compared to the individual semiconductors, extending their lifetime. This phenomenon is guaranteed by the electron transfer from the ZnO conduction band to the empty localized 4f levels of Ce^4+^, which is then reduced to Ce^3+^. Moreover, the presence of the 4f levels also extends the photons absorption to the visible range. Finally, the impregnation with Cu_2_O further increases the visible light collection, as the resulting band gap has an energy width of 2.4 eV, which corresponds to radiation with a wavelength of 520 nm. Moreover, the copper(I) oxide is characterized by a more negative flat band potential of the conduction band (CB) compared to bare ZnO which makes it more suitable for reduction processes. Within this scenario [30,31,32], the high oxidation potential of the photogenerated reactive species may be exploited for CECs and odorous substances abatement, while the reduction of Hg(II) species by the photogenerated electrons may occur, with the subsequent metal deposition on the photocatalyst surface. Therefore, the photodegradation of aqueous solutions containing the following was investigated by means of the developed materials: (i) four CECs with diverse characteristics and applications, namely diclofenac, which is a commonly used analgesic, benzotriazole, which is mostly used as corrosion inhibitor, bisphenol A, an additive in the production of plastics and the antibiotic sulfamethoxazole [33,34,35,36,37]; (ii) HgCl_2_; and (iii) geosmin and MIB as representatives of odorous molecules.

## 2. Results and Discussion

### 2.1. Mercury Removal

**Figure 1:**
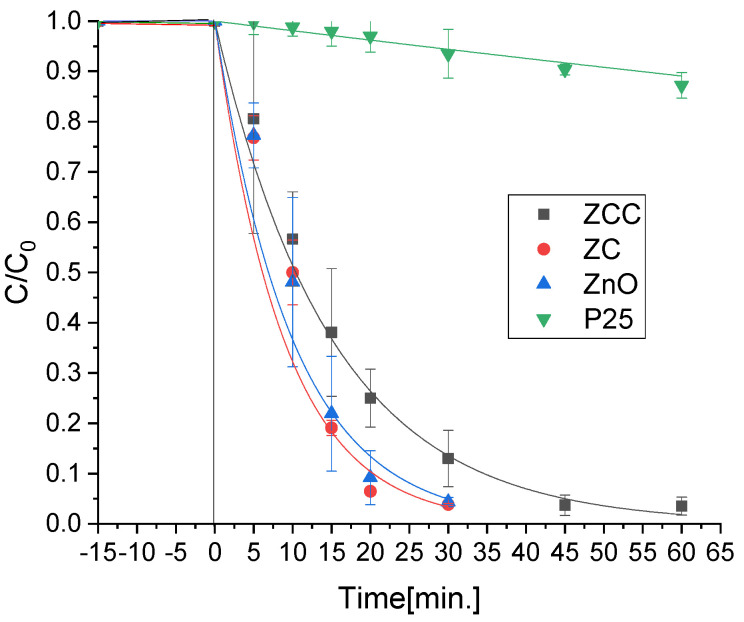
Mercury removal using the synthetized photocatalysts and the benchmark Evonik P25 under irradiation in a solar light simulator, after N_2_ stripping and 15 min of equilibration in the dark.

The mercury solution was firstly equilibrated in the dark for 15 min during which a negligible adsorption on the catalysts was observed. Then, mercury removal was evaluated using a sun simulator with a cut-off filter at 340 nm in the presence of the prepared photocatalysts (Figure 1).

All the synthesized materials showed a removal efficiency higher than the benchmark TiO_2_-P25, which under these experimental conditions was not able to lead to the metal deletion. By contrast, the ZnO-based photocatalysts managed to achieve almost the complete mercury abatement from the solution in less than 1 h. Indeed, using ZnO and ZC, 80% of the Hg(II) was removed after only 15 min.

Mercury removal was also tested at different values of pH (4, natural pH, which was equal to 6, and 8) and catalyst loads (0.2, 0.5 and 1 g/L). The best performance was obtained at natural pH in the presence of the highest quantity of the catalyst.

Experiments were repeated without nitrogen stripping, but it was observed that the presence of oxygen severely reduced the photocatalytic activity compared to anoxic conditions.

### 2.2. CECs Degradation

Four contaminants of emerging concern, namely benzotriazole (BTA), bisphenol A (BPA), diclofenac (DIC) and sulfamethoxazole (SMZ), were treated in the presence of the developed materials. Preliminarily, dark adsorption toward the considered molecules and direct photolysis experiments were carried out with all the materials. Both contributions were found to be negligible over the examined time window.

**Figure 2:**
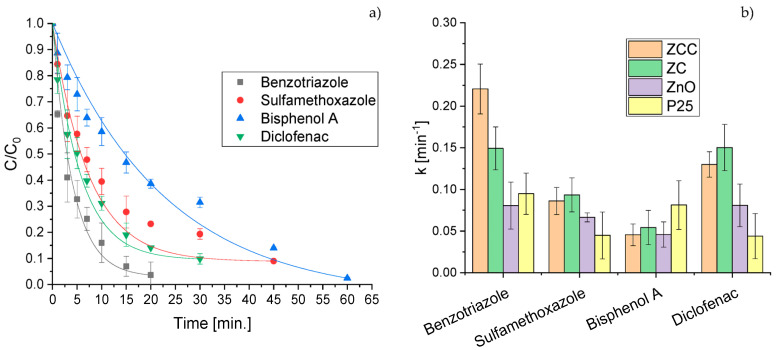
Degradation profiles of CECs obtained using ZCC photocatalyst (**a**) and kinetic constants estimated for CECs abatement reaction (**b**).

The degradation results obtained for the studied CECs in the presence of the different synthesized photocatalysts and the TiO_2_ P25 benchmark are displayed in Figure 2. The degradation profile obtained for ZCC is shown as example in Figure 2a. In all cases, the degradation process can be fitted to a pseudo first-order kinetics, with the calculated constants displayed in Figure 2b.

Except for bisphenol A for which P25 showed a greater photodegradation efficiency, ZnO-based materials exhibited higher photoactivity than P25, significantly enhanced by the incorporation of CeO_2_ and Cu_2_O. In fact, with ZCC a 50% abatement of all the contaminants was attained after 15 min.

The results obtained with ZC and ZCC were comparable, with BTA as the only exception, for which double heterojunction with the addition of CeO_2_ and Cu_2_O led to a significantly faster degradation. Further tests were also performed on other CECs and their degradation profiles (Figure S5) confirmed the greater photodegradation performance of ZCC compared with other ZnO-based materials and in three out of four cases also with respect to the benchmark P25. Recyclability tests of the photocatalysts were performed using phenol as the model pollutant (Figure S6) demonstrating that the materials can be reused, albeit with a slight decrease in the degradative efficiency.

The results of experiments carried out in the presence of scavengers are shown in Figure S7. The degradation mechanism changed depending on the investigated contaminant and the employed photocatalyst. In the case of benzotriazole, the results obtained were consistent for all of the photocatalysts: the addition of tert-butyl alcohol, scavenger of **^·^**OH, did not noticeably affect the degradation of the molecule while **^·^**O_2_^-^ seemed to play a more important role; once p-benzoquinone was added, the degradation rate decreased from 100% in the absence of scavengers to 45% after one hour of irradiation. Sulfamethoxazole was significantly less degraded by ZCC in the presence of tert-butyl alcohol, suggesting that hydroxyl radicals are majorly involved; in the case of ZC and ZnO, on the other hand, the addition of scavengers did not greatly decrease the degradation rate, suggesting that another ROS such as singlet oxygen could drive the degradation.

The photodegradation of bisphenol A and diclofenac was meaningfully affected by the presence of p-benzoquinone, with a degradation rate close to 0 after one hour, as such, superoxide radicals played a key role in the degradation mechanism while hydroxyl radicals were not involved at all in the degradation of diclofenac by ZC and ZnO and only marginally in the case of ZCC and in the degradation of bisphenol A by the prepared materials.

At the same time, we performed supplementary experiments with the EPR spin label technique using DMPO as the spin trap for the detection of **^·^**OH radicals. The results are reported in the Supplementary Materials Figure S8 and are in agreement with the experiments carried out in the presence of scavengers. The most active sample in the production of **^·^**OH radical was the ternary sample ZCC.

### 2.3. Geosmin and MIB Degradation

**Figure 3:**
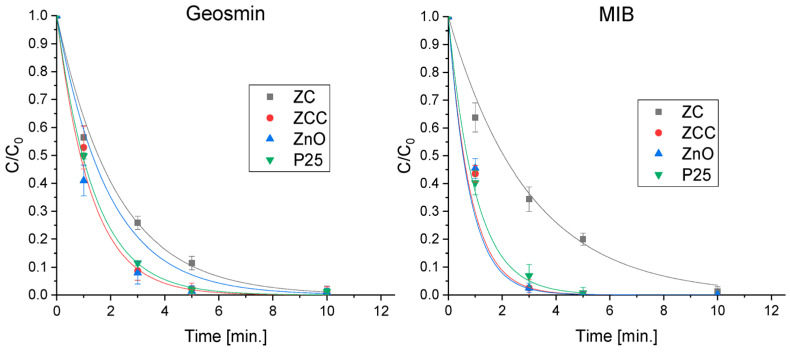
Degradation profiles of geosmin and MIB obtained using synthesized materials and the benchmark P25.

The degradation efficiency towards geosmin and MIB was assessed with the synthetized materials and with the benchmark P25. The removal of the two molecules was extremely fast for all the photocatalysts used with the complete disappearance of the organic molecules in less than 10 min, as shown in Figure 3. In this case, the heterojunction impregnated with Cu_2_O showed a better photoactivity compared to ZC and pristine ZnO, leading to the abatement of geosmin in 7 min and to the degradation of MIB in 5 min.

In light of the obtained data, the higher photocatalytic efficiency of ZC and ZCC materials compared with pristine ZnO and benchmark P25 can be explained in terms of a better absorption of light in the visible region and increased charge separation and electron stabilization in the f orbitals of Ce^4+^. Hence, as shown in previous studies [30,38], the interface between ZnO and CeO_2_ allows for more photoinduced charge carriers to be stabilized in the solid due to the transfer of electrons from the valence band of ZnO to the empty and localized 4f levels of Ce^4+^, which is reduced to Ce^3+^.

In addition, the presence of Cu_2_O increases the photon absorption in the visible region due to its band gap, and previous UV–Vis analysis and EPR measurements revealed the presence of residual traces of unreduced Cu(II) dispersed on the semiconductor surface, which could play a role in photodegradation reactions.

### 2.4. Removal of the Mixture of Different Classes of Pollutants in Actual Water

**Figure 4:**
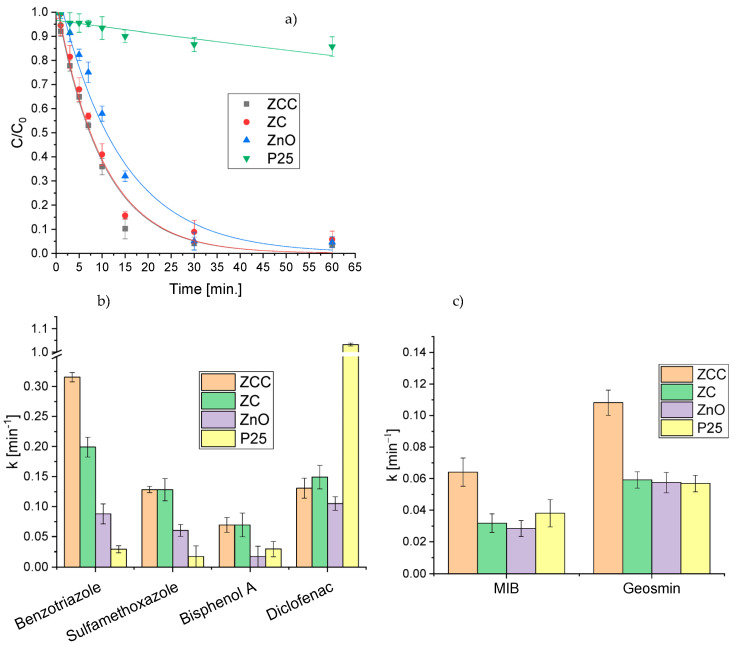
Comparison of photocatalytic mercury removal profiles (**a**) and pseudo-first order calculated kinetic constants of CECs degradation (**b**) and MIB and geosmin degradation (**c**) with the different photocatalysts in the mixture of pollutants.

In order to get experimental conditions closer to real ones, tests were carried out using a mixture of all the contaminants previously assessed individually, maintaining similar concentrations. The comparison of the performances of the photocatalysts is reported in Figure 4. Additionally, in this instance mercury removal could only be achieved through the use of ZnO-based materials but, in contrast to the results obtained in the single tests, ZnO showed a lower effectiveness than the two modified materials. As regards CECs, the comparison between the photocatalysts yielded similar results to those obtained with the single pollutants, except for diclofenac for which P25 proved to be much more effective. Overall, the better performance for the contaminants’ removal was obtained with ZCC, especially towards the odorous molecules, attaining the abatement of all pollutants within 1 h. The analysis of the kinetic constants calculated for ZCC revealed that the removal rate of Hg(II) and CECs in the mixture was even higher than that obtained for solutions with the single contaminants (see Table 1).

**Figure 5:**
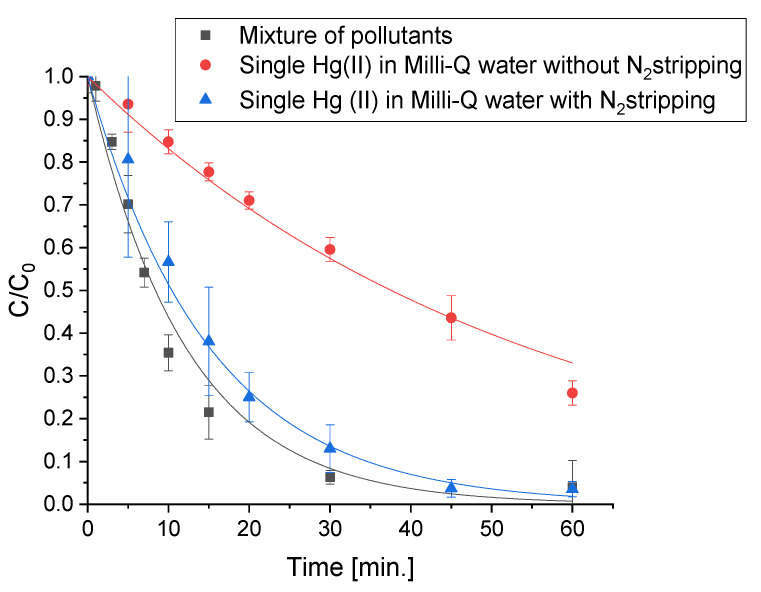
Hg(II) removal using ZCC in different working conditions.

It is noteworthy that the experiments for mercury removal performed on the single metal required the stripping with nitrogen of the solution with the aim of removing oxygen that might compete with the Hg(II) species for the photogenerated electrons. As a matter of fact, when the same test was repeated without stripping, the photocatalytic performance was clearly worse (Figure 5). However, when irradiation was carried out on the mixture of pollutants without nitrogen purging, it was observed at a faster removal of mercury than in the reductive atmosphere. This is likely caused by the lower charge recombination that takes place in the presence of the CECs and odorous molecules, since photogenerated holes should be involved in the oxidation process of the organic molecules while the electrons in the reduction of mercury.

**Figure 6:**
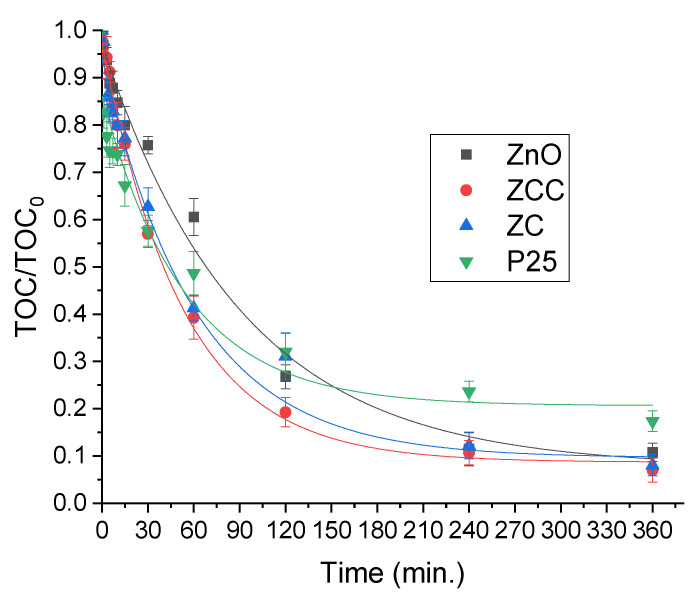
Total Organic Carbon abatement for the mixture of pollutants in the presence of the different photocatalysts.

The removal of the Total Organic Carbon (TOC) was monitored along the reaction performed with the mixture of the three classes of pollutants in Milli-Q water following a longer irradiation time for attaining the organics degradation. The results are reported in Figure 6.

The complete removal of the initial contaminants was accomplished in 60 min while, as expected, more time was needed to attain the complete mineralization in the mixture of compounds. Nevertheless, after only 1 h a 60% decrease in initial TOC was obtained using the two doped photocatalysts (ZC and ZCC) whereas a 40% reduction was achieved with the pristine ZnO and the benchmark P25, hence indicating the presence of a higher amount of remaining degradation by-products. After 6 h, the almost complete mineralization was reached with zinc oxide-based materials, while more than 20% of the organic content was still present when using P25. This is in accordance with previous studies that evidenced for instance, the slow degradation of some intermediate compounds of diclofenac, with a 90% mineralization achieved after several hours of irradiation with UV-A light using P25 [39].

**Figure 7:**
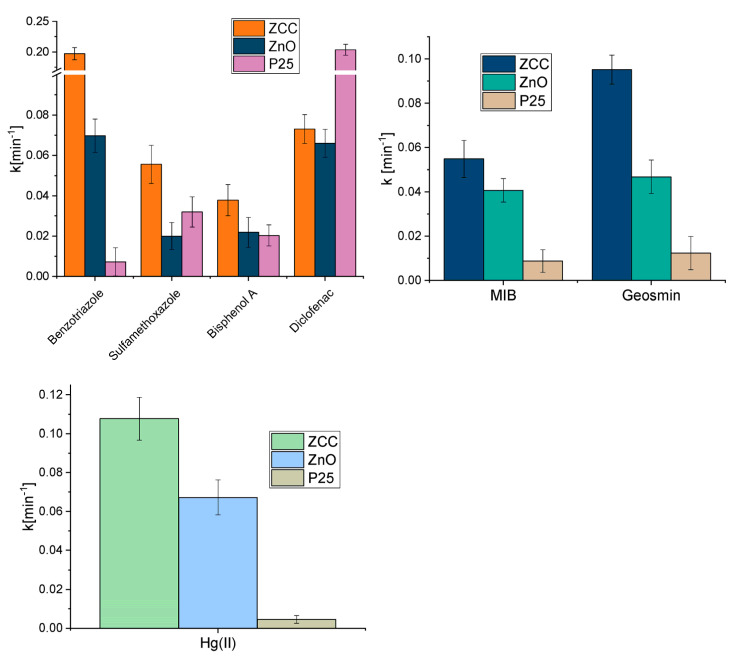
Comparison between the kinetic constants obtained using ZCC, ZnO and P25 for the abatement of the mixture of Hg(II), CECs, MIB and geosmin in aquaculture water.

The pollutants removal was then assessed in real water sampled from an aquaculture plant of koi carps, using pristine ZnO, P25 and ZCC as photocatalysts. The calculated pseudo-first order kinetic constants are displayed in Figure 7, showing that the heterojunction between ZnO and CeO_2_ and the impregnation with Cu_2_O led to a clear increase in the degradative capacity of the ZCC catalyst toward a complex mixture of pollutants in an actual matrix in respect to pristine ZnO and to a greater degradation efficiency in comparison to the benchmark P25.

However, focusing on ZCC, the kinetic constants shown in Table 1, shows the significant role of the water matrix on the photocatalytic performance. Compared to Milli-Q water, the degradation rate of odorous molecules decreased significantly in the real water, with 30 min being required for their complete removal. As regards the abatement of CECs, a decrease in the kinetic constants was registered as well, especially for diclofenac. It is probable that the cause of the longer degradation times was due to the high organic load of the water used since we detected a NPOC value equal to 15 mg/L. Indeed, as the complexity of the matrix grew, the degradation rate decreased because of the presence of dissolved organic matter which could act as a scavenger of radical intermediates. By contrast, the photocatalysts maintained good performances for mercury(II) removal and their efficiency in this case was not affected by the matrix change.

**Figure 8:**
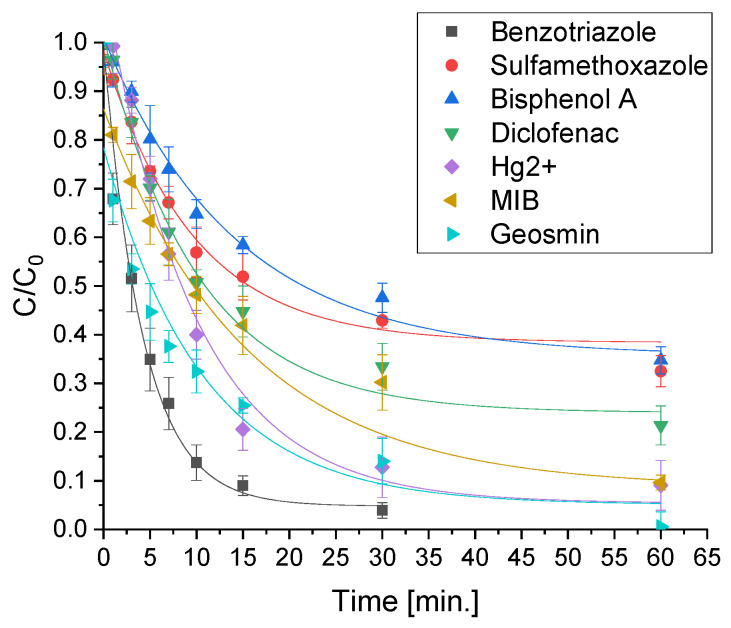
Degradation curves obtained for the mixture of contaminants using ZnO-CeO_2_-Cu_2_O as photocatalyst in aquaculture water.

As Figure 8 shows, a large amount of the pollutants were also efficiently removed in a real scenario.

## 3. Materials and Methods

### 3.1. Synthesis of the Materials

Pristine ZnO and CeO_2_/ZnO (ZC) containing 1% molar of cerium were synthetized via the hydrothermal route using the following procedure.

Zn(NO_3_)_2_ (added with CeCl_3_*7H_2_O in stoichiometric proportion in the case of the biphasic material) was solubilized in 20 mL of H_2_O in order to obtain 1M solution and NaOH 4M was added until pH 10–11 was reached. The obtained solution was transferred into a PTFE-lined stainless steel 100 mL autoclave and heated at 448 K for 12 h. The collected samples were washed and centrifuged 3 times at 6000 rpm for 10 min and left to dry in the oven at 343 K [38]. The ZnO powder was white as expected while ZC was pale yellow.

The impregnation of ZC obtained by the synthesis described above was performed according to Benedict’s reaction in which glucose reduces Cu^2+^ to Cu^+^ allowing the formation of Cu_2_O oxide.

A solution of Cu(NO_3_)_2_*3H_2_O 7.9 × 10^−3^ M was prepared and 10 mL was added with 15 mL of NaOH 2.4 × 10^−3^ M and 0.0568 g of glucose. It was placed under stirring in a water bath heated to 343 K being careful not to exceed this temperature, otherwise the precipitate could dissolve. As the reaction proceeded, a change in color was observed: blue, green, light yellow and finally intense orange.

An amount of 1 g of ZC was suspended into 20 mL of deionized H_2_O and put in an ultrasonic bath for 10 min at 333 K. The suspension was then added to the beaker containing the precipitated Cu_2_O and the mixture was left at 343 K under magnetic stirring for 10 min. Finally, the precipitate was filtered, washed with deionized H_2_O and ethanol and dried in the oven [38]. The final sample, Cu_2_O/CeO_2_/ZnO photocatalysts (ZCC), had a deep pink–orange color. Characterization of materials can be found in the literature and is reported for sake of clarity in Figures S1–S4.

### 3.2. Pollutants Removal

#### 3.2.1. Mercury Removal

Synthesized photocatalysts efficiency was tested in simulated conditions of solar irradiation. Experiments were performed using closed Pyrex cells (40 mm id × 25 mm) containing 5 mL of suspension of the catalyst (C = 0.5 g/L) and 50 mg/L of Hg(II) prepared by dissolving HgCl_2_ in Milli-Q water. The suspension was kept in the dark for 15 min under continuous stirring and then irradiated in a solar light simulator (Solarbox CO.FO.MEGRA) equipped with a 1500 W Xenon lamp and a 340 nm cut-off filter. Before irradiating, the cell was saturated with nitrogen blown through a cap provided with a pierceable septum with a stainless-steel needle. During the experiment, samples were taken at different time intervals (t_0_ before adsorption in the dark, 15 min after adsorption, t_0_ before irradiation, and after 5, 10, 15, 20, 30, 45, 60, 90, and 120 min of irradiation) and filtered through 0.45 µm PTFE membranes to remove the suspended catalyst before being analyzed. Evonik P25 titanium dioxide was used as benchmark. The mercury concentration was determined by Inductively Coupled Plasma Atomic Emission Spectrometry (ICP-OES) or by Cold Vapor Atomic Fluorescence Spectroscopy (CVAFS) according to the concentrations to be measured and the sensitivity of the instrument.

#### 3.2.2. CECs Degradation

The degradation tests on benzotriazole, bisphenol A, diclofenac and sulfamethoxazole were performed at natural pH (pH = 7.1) in the presence of the catalyst at 0.2 g/L. The concentration of each compound was 4 mg/L. All the experiments were performed in Pyrex glass cells kept under magnetic stirring and filled with 5 mL of sample and catalyst suspension. The cells were irradiated in a Solarbox equipped with a 340 nm cut-off filter. The samples were filtered through a 0.45 μm PTFE membrane and the photodegradation process was followed over time (sampling times: 0, 1, 3, 5, 7, 10, 15, 30, 60, and 120 min). All the synthetized materials were tested and Evonik P25 titanium dioxide was used as benchmark. Other pollutants namely phenol, carbamazepine, tolytriazole, and ofloxacin were preliminarily tested under the same conditions, and employing phenol as a model pollutant, the possibility of reusing the photocatalysts was verified by recovering them from the filter after utilization and using them for a second and third cycle of degradation. Analyses were performed with a Merck-Hitachi HPLC system equipped with a L-6200A Intelligent Pump, a L-4200 UV–VIS Detector and a six-way Rheodyne valve injection system. The detection wavelength was set at 220 nm and a gradient elution was performed with a mixture of phosphoric acid solution at pH 2.8 (A) and acetonitrile (B) at a flow rate of 1 mL/min in accordance with the following program:  75:25 A/B for 3 min; 75:25 to 65:35 over 4 min; 65:35 to 40:60 over 8 min; 40:60 for 5 min; 40:60 to 75:25 over 0.5 min; 75:25 for 5 min.

The retention times were 2.96, 5.35, 14.08 and 18.20 min for benzotriazole, sulfamethoxazole, bisphenol A and diclofenac, respectively.

Moreover, in order to further comprehend the mechanism of degradation of CECs by synthetized photocatalysts, experiments were repeated using two scavengers of ROS. Specifically, tert-Butyl alcohol in concentration equal to 10 mM was added as a scavenger of hydroxyl radicals and p-Benzoquinone 10 mM as a scavenger of superoxide radicals.

#### 3.2.3. Geosmin and MIB Degradation

ZnO-based photocatalysts were also tested on aqueous solutions of geosmin and MIB (C = 5 µg/L of each compound) following the same procedure described in the previous paragraph. A headspace solid phase micro extraction (HS-SPME) method combined with gas chromatography–mass spectrometry (GC–MS) was developed for the analysis. A medium polar SPME fiber Divinylbenzene/Carboxen/Polymethylsiloxane (DVB/CAR/PDMS) assembled on a SPME holder was used and extraction conditions were fine-tuned. The optimized method involved the use of 10 mL of sample inserted in a proper vial with 3 g of NaCl added to favor the salting out. The vial was then capped with a screw cap with a silicone/PTFE septum, stirred at 300 rpm and heated in a thermostatic bath at 40 °C for 10 min (equilibration time). Then, the SPME fiber was pierced through the septum and exposed in the headspace of the vial for 25 min.

Fiber exposure time and temperature were optimized: fiber exposure times in the headspace of 10, 15, 20, 25 and 30 min were evaluated by observing the variation of the peak signal of a standard solution of geosmin and 2-MIB according to exposure time. The best signal was obtained after 25 min and it remained stable at longer times. The sample temperature was varied from 20 to 80 °C and the optimum value was found to be 40 °C.

Finally, the fiber was inserted into the GC–MS injector and the analytes were thermally desorbed. The GC–MS system consisted of an Agilent 8860 GC System, an Agilent 5190-4048 Ultra Inert liner Straight injector, a SPME taper 0.75 mm, an Agilent J&W GC Columns 30 m × 250 μm × 025 μm and an Agilent 5977B GC/MSD GC Mass Detector. The analyses were conducted setting the He flow at 1.5 mL/min, the liner temperature at 270 °C, the MS transfer line temperature at 300 °C, the MS source temperature at 300 °C and the quadrupole temperature at 150 °C. The injection was performed through the pulse–splitless method, which injects the sample through a pressure pulse into the liner chamber with a set pressure of 30 psi, and a purge–flow split vent of 40 mL/min to clean the chamber after injection.

The chromatographic run consisted of a starting temperature of 333 K maintained for 1 min, then the temperature was increased by 10 °C/min until 523 K for a total GC runtime of 20 min. The retention times were 7.00 min for 2-MIB and 10.53 min for geosmin. The analysis was performed in Single Ion Monitoring (SIM) mode and target m/z ratios 112.0 and 95.1 were used for quantification of geosmin and 2-MIB, respectively. The ratios of 111.0, 125.0 and 182.0 m/z were used for analyte confirmation of geosmin while 108.1 and 135.1 were utilized as qualifier for 2-MIB. Quantification of peak areas was performed using the Agilent MassHunter Quantitative Analysis program.

#### 3.2.4. Removal of the Different Classes of Pollutants in Mixture and in Actual Water

After performing experiments to evaluate the efficiency of the prepared photocatalyst on solutions individually containing the three classes of analytes, tests were also carried out with a mixture of them.

Concentrations of the various analytes were unchanged with respect to the single tests while 0.2 g/L was used as the photocatalysts load. The photocatalytic tests were initially carried out on the mixture using Milli-Q as a matrix and subsequently repeated using water from an aquaculture plant where koi carps are grown. Water was sampled using a sample bottle and collected at a depth of 5–10 cm at each of the 5 sampling points of the aquaculture pond. It was stored into amber glass bottles of 1 L capacity with stoppers or Teflon-lined screw caps until the arrival at the laboratory.

After sampling, the water was immediately filtered with a 0.7 µm glass microfibre filter 698 supplied by VWR International and stored in darkness at 277 K.

Samples deriving from the photocatalytic experiments on the mixture were analysed as described in the above paragraphs. In the samples prepared in Milli-Q water, the Total Organic Carbon (TOC) was measured at different reaction times using a TOC-VCSH analyzer.

## 4. Conclusions

The abatement from aqueous solution of representative compounds of three class of relevant pollutants, i.e., organic molecules belonging to pollutants included in the category of contaminants of emerging concern, (i.e., benzotriazole, bisphenol A, diclofenac and sulfamethoxazole), odorous molecules responsible for poor organoleptic characteristics of aquaculture fish, i.e., geosmin and 2-methylisoborneol, and Hg(II) was efficiently attained using ZnO-based photocatalysts. In particular, the hydrothermal synthesis of the ZnO-CeO_2_ heterojunction and subsequent impregnation with Cu_2_O nanoparticles resulted in an extremely effective material, which allowed the photocatalytic degradation of a wide range of pollutants including recalcitrant molecules by means of a sustainable energy source such as solar radiation in less than one hour. The photocatalyst Cu_2_O/CeO_2_/ZnO showed a slightly higher efficiency than the heterojunction without impregnation, pristine zinc oxide and the benchmark P25 toward the degradation of the organic contaminants and a significantly higher efficiency in mercury removal than TiO_2_ P25. In addition, the removal of the target compounds from the mixture of CECs, odorous molecules, and Hg(II) was successfully obtained, thus, showing that the efficiency of the photocatalyst was not adversely affected; conversely, the simultaneous presence of organic pollutants and the metal led to higher kinetic constants of the photocatalytic reactions, probably because of the more efficient charge separation. Excellent results were also achieved when using actual water sampled from an aquaculture farm as a matrix, boding well for future use on the field.

## Figures and Tables

**Table 1:** Comparison between the kinetic constants obtained with the ZCC photocatalyst for the removal of the evaluated pollutants, individually and in a mixture of them in two different matrices: Milli-Q and real aquaculture water.

Matrix	Composition	k [min ^−1^]						
		BTA	SMZ	BPA	DIC	Hg(II)	MIB	Geosmin
Milli-Q water	Single class	0.22	0.09	0.05	0.13	0.07	1.68	0.80
Milli-Q water	Mixture	0.32	0.13	0.07	0.13	0.10	0.06	0.11
Aquaculture	Single class	0.17	0.05	0.04	0.05	0.11	0.06	0.09
Aquaculture	Mixture	0.20	0.06	0.04	0.07	0.10	0.05	0.10

## Data Availability

Data, associated metadata, and calculation tools are available from the corresponding author.

## Supplementary Materials

The following supporting information can be downloaded at: https://www.mdpi.com/article/10.3390/molecules28062650/s1, Figure S1: XRD pattern of ZnO, CeO_2_-ZnO (ZC) and Cu_2_O-CeO_2_-ZnO (ZCC); Figure S2: TEM image of (A) pure ZnO and (B) ZC. Images of ZCC sample are not available because the sample holder of our microscope is made in copper so it is not possible to measure sample containing copper; Figure S3: Adsorption spectra of ZnO, ZC and ZCC samples; Figure S4: XPS deconvoluted spectrum of the Ce 3d region for the sample ZC; Figure S5: Degradation profiles of Phenol, Carbamazepine, Tolyltriazole, Ofloxacin obtained using ZCC, ZC, ZnO and reference P25 photocatalyst; Figure S6: Photocatalysts recyclability test on phenol degradation; Figure S7: The degradation percentage of CECs obtain using ZCC, ZC and ZnO photocatalysts after one hour of irradiation in the presence of scavengers t-Butyl alcohol and p-Benzoquinone of hydroxyl and superoxide radicals respectively; Figure S8: Integrated intensities of the EPR spectra as a function of irradiation time of the DMPO/OH**·** adduct produced by irradiation of an aqueous suspension of the samples ZnO, ZC and ZCC samples with UV visible light (λ > 360nm).

## References

[1] World Health Organization, United Nations Children’s Fund (2019). Progress on Household Drinking Water, Sanitation and Hygiene 2000–2017: Special Focus on Inequalities.

[2] Pörtner H.-O., Roberts D.C., Tignor M., Poloczanska E.S., Mintenbeck K., Alegría A., Craig M., Langsdorf S., Löschke S., Möller V. (2022). IPCC, 2022: Climate Change 2022: Impacts, Adaptation, and Vulnerability. Contribution of Working Group II to the Sixth Assessment Report of the Intergovernmental Panel on Climate Change.

[3] Sanchez-Salas J., Flores D., Bandala E. (2018). Water recalcitrant contaminants: Sources, assessment and remediation. Wastewater and Water Contamination: Sources, Assessment and Remediation.

[4] Warren N., Allan I.J., Carter J.E., House W.A., Parker A. (2003). Pesticides and other micro-organic contaminants in freshwater sedimentary environments—A review. Appl. Geochem..

[5] Alharbi O.M.L., Basheer A.A., Khattab R.A., Ali I. (2018). Health and environmental effects of persistent organic pollutants. J. Mol. Liq..

[6] Golovko O., Örn S., Sörengård M., Frieberg K., Nassazzi W., Lai F.Y., Ahrens L. (2021). Occurrence and removal of chemicals of emerging concern in wastewater treatment plants and their impact on receiving water systems. Sci. Total Environ..

[7] Gogoi A., Mazumder P., Tyagi V.K., Tushara Chaminda G.G., An A.K., Kumar M. (2018). Occurrence and fate of emerging contaminants in water environment: A review. Groundw. Sustain. Dev..

[8] Strungaru S.-A., Nicoara M., Teodosiu C., Baltag E., Ciobanu C., Plavan G. (2018). Patterns of toxic metals bioaccumulation in a cross-border freshwater reservoir. Chemosphere.

[9] González-Rubio S., Ballesteros-Gómez A., Asimakopoulos A.G., Jaspers V.L.B. (2021). A review on contaminants of emerging concern in European raptors (2002–2020). Sci. Total Environ..

[10] Little D.C., Newton R.W., Beveridge M.C.M. (2016). Aquaculture: A rapidly growing and significant source of sustainable food? Status, transitions and potential. Proc. Nutr. Soc..

[11] FAO (2020). The State of World Fisheries and Aquaculture 2020. Sustainability in Action.

[12] Lindholm-Lehto P.C., Vielma J. (2018). Controlling of geosmin and 2-methylisoborneol induced off-flavours in recirculating aquaculture system farmed fish—A review. Aquac. Res..

[13] Howgate P. (2004). Tainting of farmed fish by geosmin and 2-methyl-iso-borneol: A review of sensory aspects and of uptake/depuration. Aquaculture.

[14] Calza P., Sakkas V.A., Medana C., Baiocchi C., Dimou A., Pelizzetti E., Albanis T. (2006). Photocatalytic degradation study of diclofenac over aqueous TiO2 suspensions. Appl. Catal. B Environ..

[15] Ollis D.F., Pelizzetti E., Serpone N. (1991). Photocatalyzed destruction of water contaminants. Environ. Sci. Technol..

[16] Musial J., Mlynarczyk D.T., Stanisz B.J. (2023). Photocatalytic degradation of sulfamethoxazole using TiO(2)-based materials—Perspectives for the development of a sustainable water treatment technology. Sci. Total Environ..

[17] Shi W., Liu Y., Sun W., Hong Y., Li X., Lin X., Guo F., Shi J. (2022). Improvement of synergistic effect photocatalytic/peroxymonosulfate activation for degradation of amoxicillin using carbon dots anchored on rod-like CoFe_2_O_4_. Chin. J. Chem. Eng..

[18] Guo F., Chen Z., Shi Y., Cao L., Cheng X., Shi W., Chen L., Lin X. (2022). A ragged porous hollow tubular carbon nitride towards boosting visible-light photocatalytic hydrogen production in water and seawater. Renew. Energy.

[19] Shi W., Sun W., Liu Y., Zhang K., Sun H., Lin X., Hong Y., Guo F. (2022). A self-sufficient photo-Fenton system with coupling in-situ production H_2_O_2_ of ultrathin porous g-C3N4 nanosheets and amorphous FeOOH quantum dots. J. Hazard. Mater..

[20] López-Muñoz M.J., Aguado J., Arencibia A., Pascual R. (2011). Mercury removal from aqueous solutions of HgCl2 by heterogeneous photocatalysis with TiO2. Appl. Catal. B Environ..

[21] Chowdhury P., Elkamel A., Ray A. (2014). Photocatalytic processes for the removal of toxic metal ions. Heavy Metals in Water: Presence, Removal and Safety.

[22] Folli A., Bloh J.Z., Strøm M., Pilegaard Madsen T., Henriksen T., Macphee D.E. (2014). Efficiency of Solar-Light-Driven TiO2 Photocatalysis at Different Latitudes and Seasons. Where and When Does TiO2 Really Work?. J. Phys. Chem. Lett..

[23] Lee K.M., Lai C.W., Ngai K.S., Juan J.C. (2016). Recent developments of zinc oxide based photocatalyst in water treatment technology: A review. Water Res..

[24] Samadi M., Zirak M., Naseri A., Khorashadizade E., Moshfegh A.Z. (2016). Recent progress on doped ZnO nanostructures for visible-light photocatalysis. Thin Solid Film..

[25] Khaki M.R.D., Shafeeyan M.S., Raman A.A.A., Daud W.M.A.W. (2017). Application of doped photocatalysts for organic pollutant degradation—A review. J. Environ. Manag..

[26] Liu X., Iocozzia J., Wang Y., Cui X., Chen Y., Zhao S., Li Z., Lin Z. (2017). Noble metal–metal oxide nanohybrids with tailored nanostructures for efficient solar energy conversion, photocatalysis and environmental remediation. Energy Environ. Sci..

[27] He X., Zhang C. (2019). Recent advances in structure design for enhancing photocatalysis. J. Mater. Sci..

[28] Wang Y., Wang Q., Zhan X., Wang F., Safdar M., He J. (2013). Visible light driven type II heterostructures and their enhanced photocatalysis properties: A review. Nanoscale.

[29] Cerrato E., Paganini M.C. (2020). Mechanism of visible photon absorption: Unveiling of the C3N4–ZnO photoactive interface by means of EPR spectroscopy. Mater. Adv..

[30] Cerrato E., Calza P., Cristina Paganini M. (2022). Photocatalytic reductive and oxidative ability study of pristine ZnO and CeO2-ZnO heterojunction impregnated with Cu2O. J. Photochem. Photobiol. A Chem..

[31] Cerrato E., Gionco C., Paganini M.C., Giamello E., Albanese E., Pacchioni G. (2018). Origin of Visible Light Photoactivity of the CeO2/ZnO Heterojunction. ACS Appl. Energy Mater..

[32] Calza P., Gionco C., Giletta M., Kalaboka M., Sakkas V.A., Albanis T., Paganini M.C. (2017). Assessment of the abatement of acelsulfame K using cerium doped ZnO as photocatalyst. J. Hazard. Mater..

[33] World Health Organization, Food and Agriculture Organization (2012). Toxicological and Health Aspects of Bisphenol A: Report of Joint FAO/WHO Expert Meeting 2–5 November 2010 and Report of Stakeholder Meeting on Bisphenol A, 1 November 2010 Ottawa, Canada.

[34] Cantwell M.G., Sullivan J.C., Burgess R.M., Zeng E.Y. (2015). Chapter 16—Benzotriazoles: History, Environmental Distribution, and Potential Ecological Effects. Comprehensive Analytical Chemistry.

[35] Careghini A., Mastorgio A.F., Saponaro S., Sezenna E. (2015). Bisphenol A, nonylphenols, benzophenones, and benzotriazoles in soils, groundwater, surface water, sediments, and food: A review. Environ. Sci. Pollut. Res..

[36] Sathishkumar P., Meena R.A.A., Palanisami T., Ashokkumar V., Palvannan T., Gu F.L. (2020). Occurrence, interactive effects and ecological risk of diclofenac in environmental compartments and biota—A review. Sci. Total Environ..

[37] Dantas R.F., Contreras S., Sans C., Esplugas S. (2008). Sulfamethoxazole abatement by means of ozonation. J. Hazard. Mater..

[38] Cerrato E., Rebolini E., Fabbri D., Calza P., Paganini M.C. (2021). Ternary systems based on ZnO/CeO_2_/Cu_2_O for the degradation of phenol and carbamazepine. J. Alloys Compd..

[39] Moctezuma E., Leyva E., Lara-Pérez C., Noriega S., Martínez-Richa A. (2020). TiO_2_ Photocatalytic Degradation of Diclofenac: Intermediates and Total Reaction Mechanism. Top. Catal..

