# Supplementary material for: Mercury and Organic Pollutants Removal from Aqueous Solutions by Heterogeneous Photocatalysis with ZnO-Based Materials
Source: Molecules. 2023 Mar 15;28(6):2650. doi: 10.3390/molecules28062650 (PMC10051193; doi:10.3390/molecules28062650)
Supplement: Supplementary file 1 [file molecules-28-02650-s001.zip › molecules-2163736-SI.pdf]

Supplementary material

Mercury and organic pollutants removal from aqueous solutions by heterogeneous photocatalysis with ZnO–based materials

Elisa Gaggero[1], María José López-Muñoz[2], Maria Cristina Paganini[1], Amaya Arencibia[3], Stefano Bertinetti[1], Nieves Fernández de Paz[4], Paola Calza[1].

1 Department of Chemistry, Università degli Studi di Torino, 10125 Torino, Italy;
2 Departamento de Tecnología Química y Ambiental, ESCET, Universidad Rey Juan Carlos, 28933 Móstoles, Madrid, Spain;
3 Departamento de Tecnología Química, Energética y Mecánica, ESCET, Universidad Rey Juan Carlos, 28933 Móstoles, Madrid, Spain;
4 Metrohm Hispania, 28044 Madrid, Spain;
* Correspondence: elisa.gaggero@unito.it (E.G.); paola.calza@unito.it (P.C.)

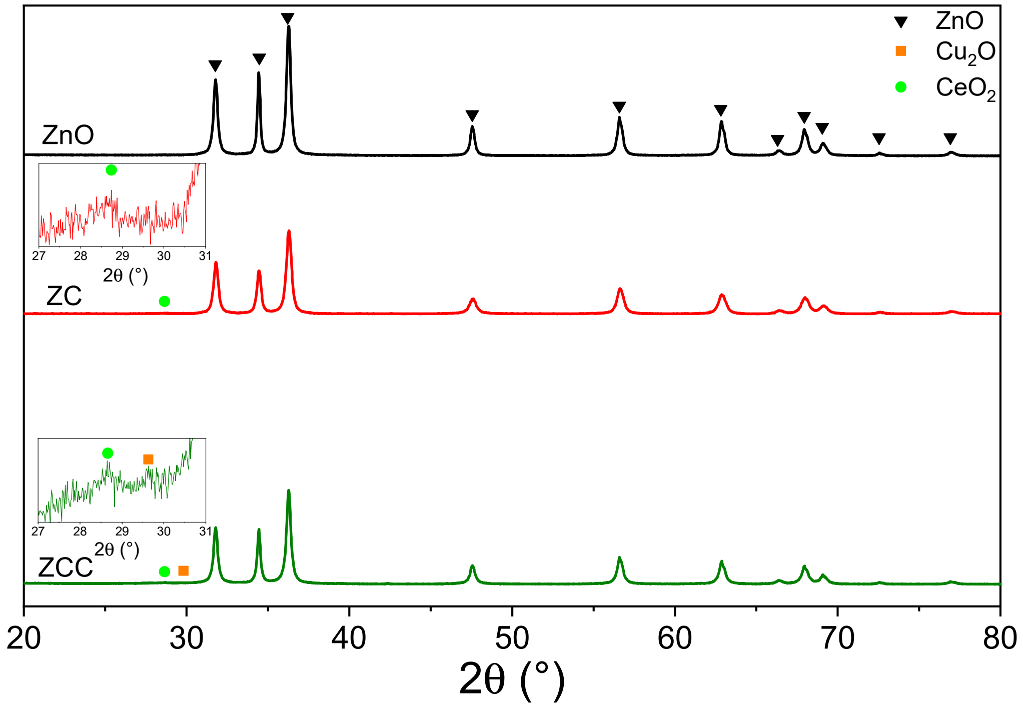

Figure S1: XRD pattern of ZnO, CeO_2–ZnO (ZC) and Cu_2O–CeO_2–ZnO (ZCC)

In Figure S1 XRD pattern of the three prepares samples are reported. The main pattern is due to the wurtzitic structure of ZnO but it is also possible to observe two weak peaks due to the presence of CeO_2 in the sample ZC and Cu_2O in the sample ZCC [E. Cerrato, C. Gionco, M. C. Paganini and E. Giamello, J. Phys. Condens. Matter., 2017, 29, 1-7; T. D. Golden, M. G. Shumsky, Y. Zhou, R. A. VanderWerd, R. A. Van Leeuwen and J. A. Sweitzer, Chem. Mater., 1996, 8, 2499-2504.]

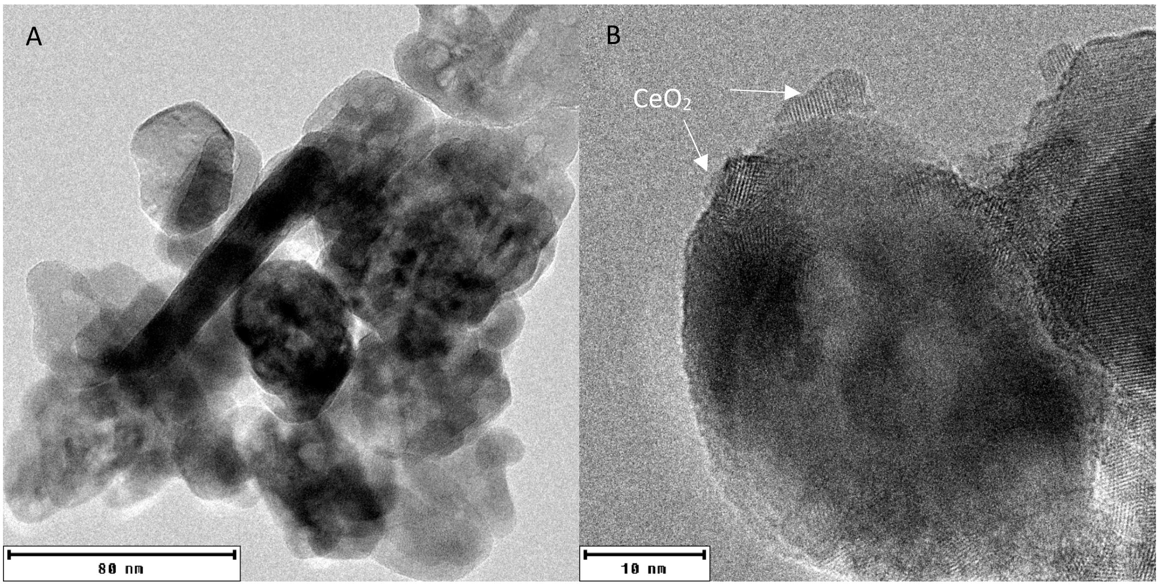

Figure S2: TEM image of A) pure ZnO and B) ZC. Images of ZCC sample are not available because the sample holder of our microscope is made in copper so it is not possible to measure sample containing copper.

For what concerns the bare sample (Fig. S2A), the particles appear with different morphologies. Most of them present platelets shape while a minor fraction shows a rounded profile. The TEM image of the doped sample (Fig.S2B) evidences the formation of CeO_2 aggregates of very small size (around 4 nm), mostly with a quasi-spherical shape, stabilized at the surface of ZnO.

EDS analysis performed on the doped sample indicates a good dispersion of the Cerium component (data not shown for brevity).

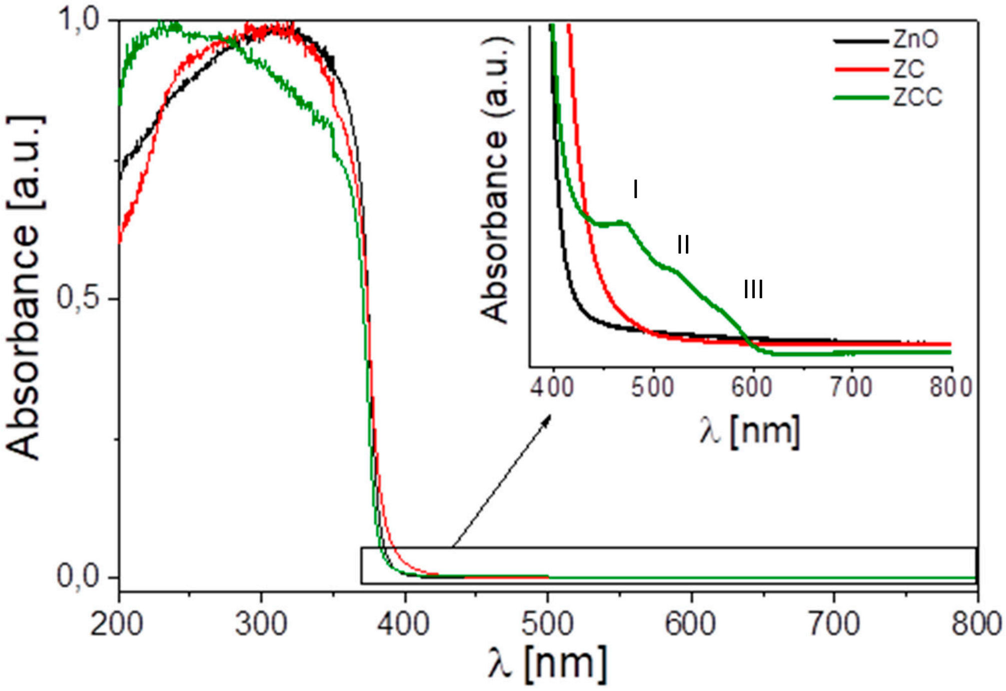

Figure S3: Adsorption spectra of ZnO, ZC and ZCC samples.

The normalize absorbance Kubelka-Munk transformed diffuse reflectance spectrum of ZnO reveals the expected absorption at wavelength lower than 400 nm, in the UV range of the electromagnetic spectrum.

Considering the interfaced material ZC, the presence of CeO_2 does not affect the band gap value; rather it introduces an additional absorption shoulder in the region 400-460 nm. The zoom in Figure S3 attests that the impregnation of copper(I) oxide greatly affected the optical absorption feature of the ZC material, adding three different contributions (I, II and III) respect to the not impregnated samples. The first one (I in the magnitude) has been suggested deriving from the interface charge transfer (IFCT) mechanism that may occur from ZnO VB to the present Cu species. The involved electron transfer, promoted by visible frequency, might partially reduce Cu_2O to metallic copper (Cu^0), similarly to what observed for TiO_2 impregnated with Cu_2O and FeO_x [I. Tamiolakis, I. T. Papadas, K. C. Spyridopoulos and G. S. Armatas, RSC Advances, 2016, 6, 54848-54855; H. Tada, Q. Jin, H. Nishijima, H. Yamamoto, M. Fujishima, S. Okuoka, T. Hattori, Y. Sumida and H. Kobayashi, Angew Chem Int Ed Engl, 2011, 50, 3501-3505]. In support of this hypothesis, the broad and low intensity contribution identified as (III) in the enlargement of Fig. S3 is ascribable to the surface plasmonic resonance (SPR) effect of metallic Cu(0) [V. Polliotto, S. Livraghi, A. Krukowska, M. V. Dozzi, A. Zaleska-Medynska, E. Selli and E. Giamello, ACS Appl Mater Interfaces, 2018, 10, 27745-27756; M. A. Sliem, T. Hikov, Z. A. Li, M. Spasova, M. Farle, D. A. Schmidt, M. Havenith-Newen and R. A. Fischer, Phys Chem Chem Phys, 2010, 12, 9858-9866]. Finally, the spectral feature labelled as (II) at almost 520 nm is undoubtedly attributed to the – VB- -CB- electronic transition in Cu_2O. [S. Banerjee, Europhys. Lett., D. Chakravorty, 52, 468-473.]

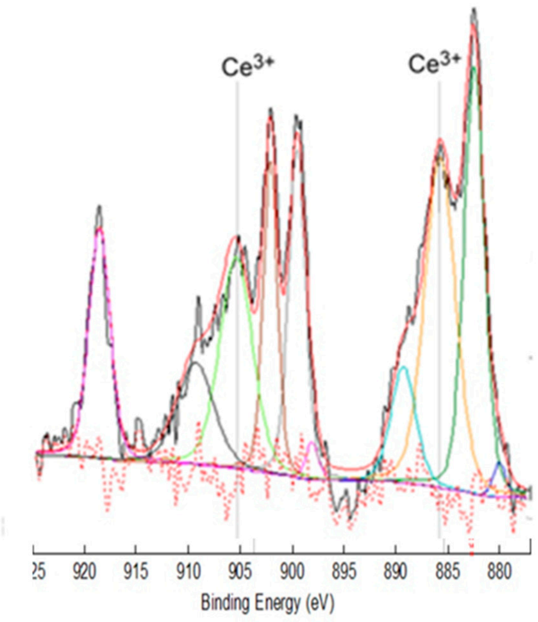

Figure S4: XPS deconvoluted spectrum of the Ce 3d region for the sample ZC.

Unfortunately for sake of time we were not able to perform XPS measurements on the ZCC sample.

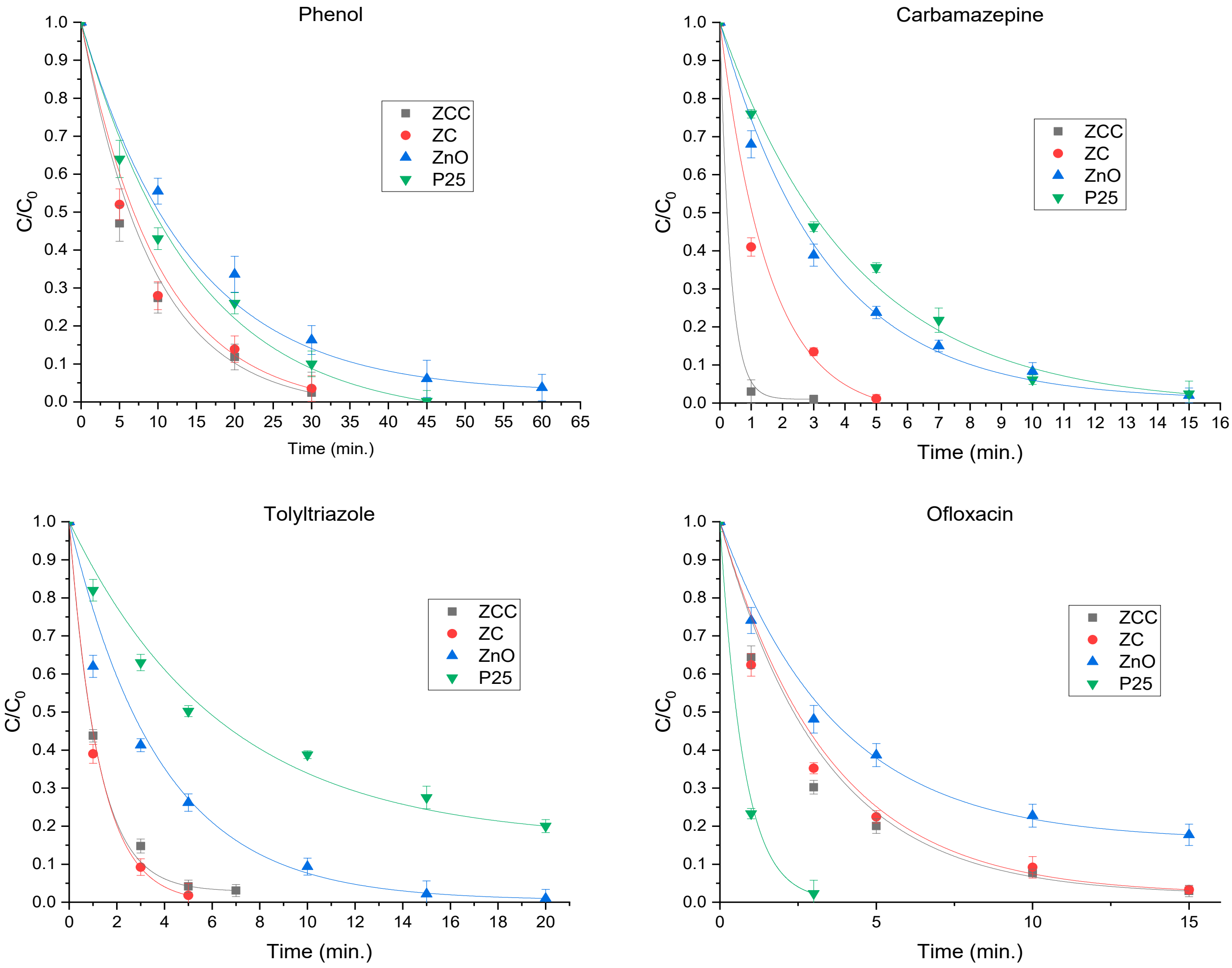

Figure S5: Degradation profiles of Phenol, Carbamazepine, Tolyltriazole, Ofloxacin obtained using ZCC, ZC, ZnO and reference P25 photocatalyst.

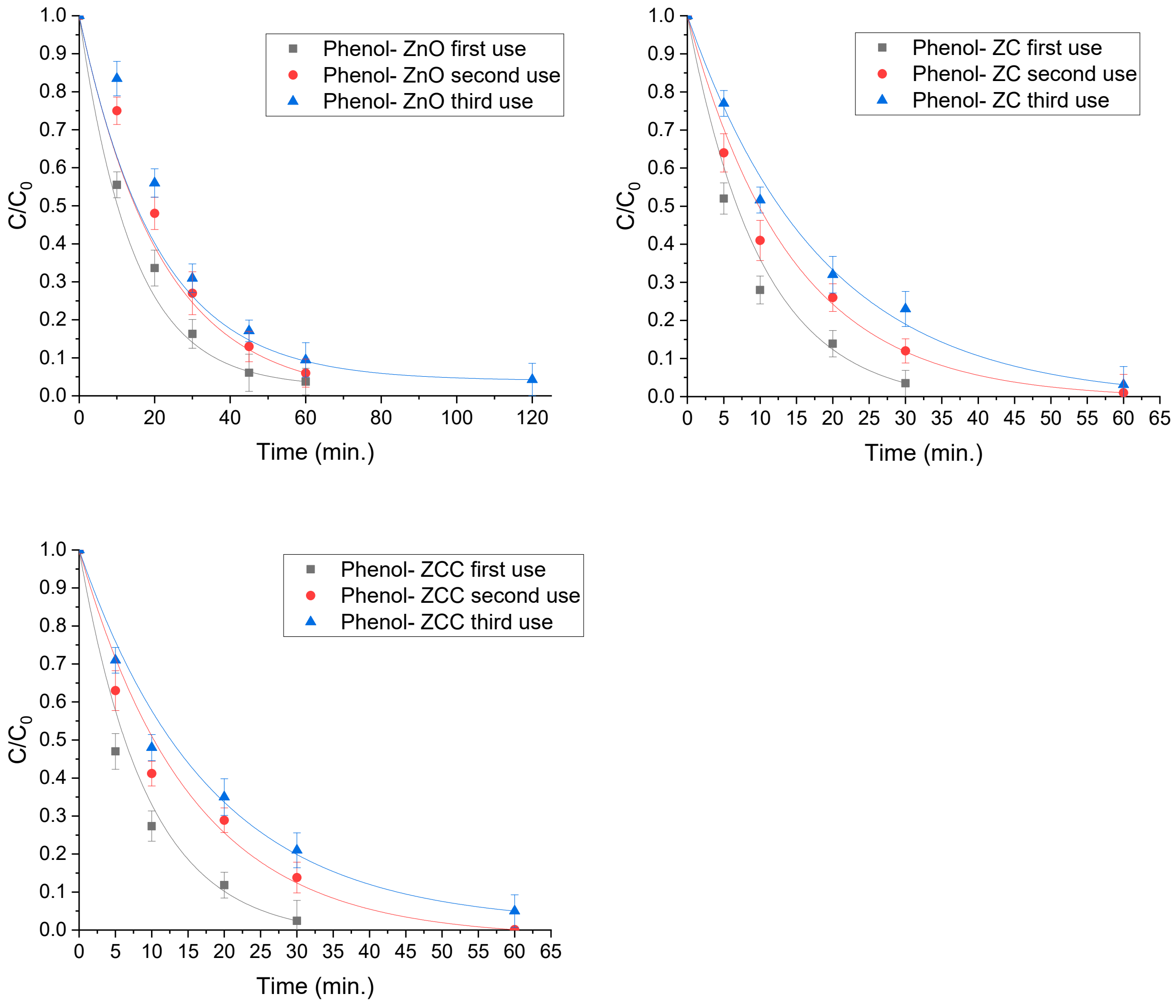

Figure S6: Photocatalysts recyclability test on phenol degradation.

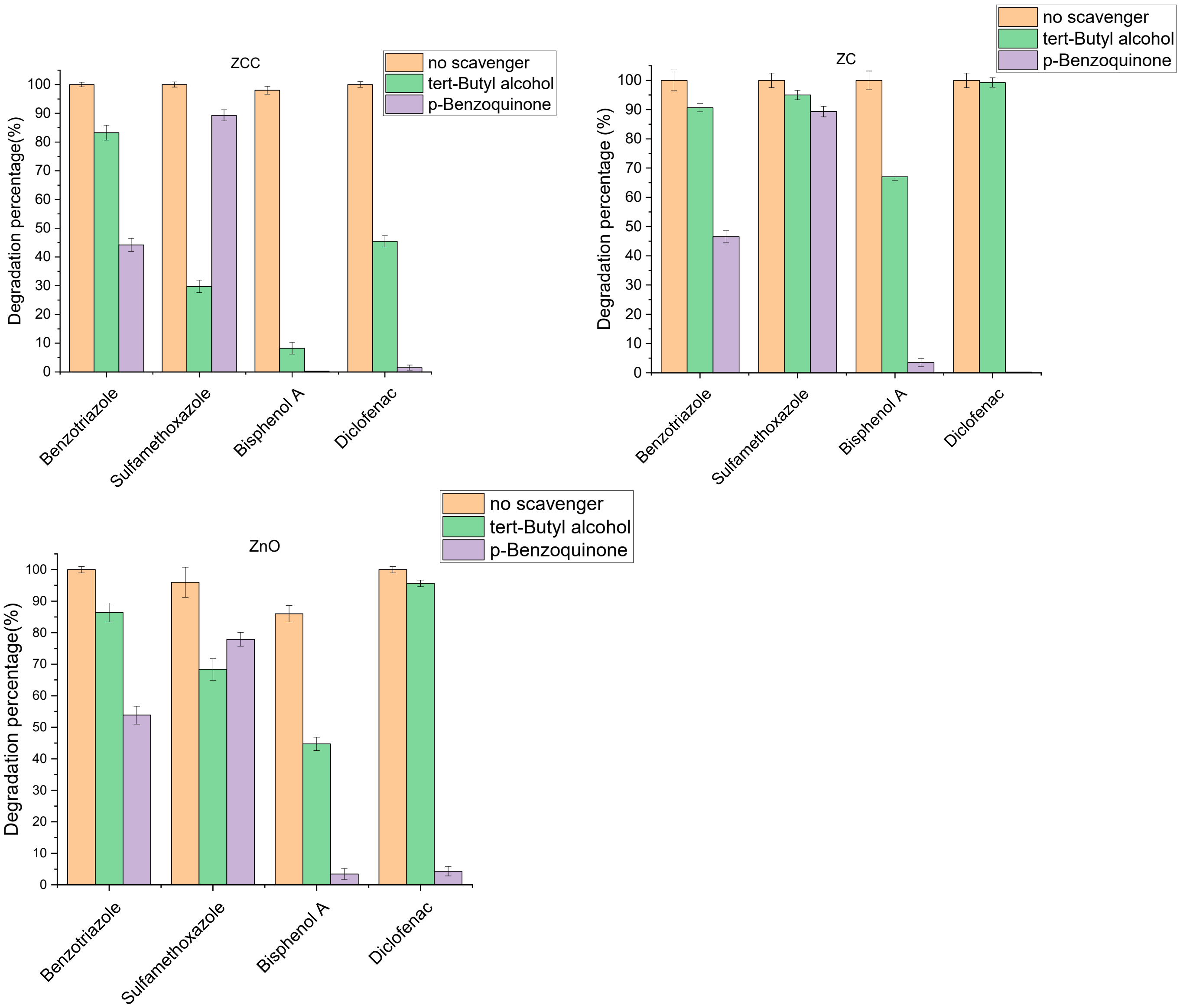

Figure S7: The degradation percentage of CECs obtain using ZCC, ZC and ZnO photocatalysts after one hour of irradiation in the presence of scavengers t-Butyl alcohol and p-Benzoquinone of hydroxyl and superoxide radicals respectively.

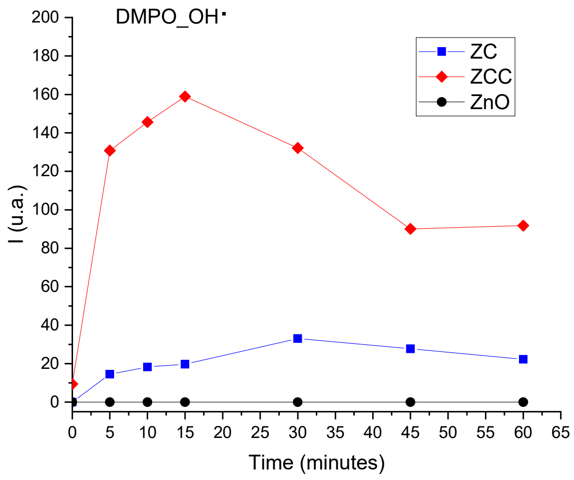

Figure S8: Integrated intensities of the EPR spectra as a function of irradiation time of the DMPO/OH· adduct produced by irradiation of an aqueous suspension of the samples ZnO, ZC and ZCC samples with UV visible light (λ>360nm).
